# Supplementary material for: β-Catenin/TCF4 Complex-Mediated Induction of the NRF3 (NFE2L3) Gene in Cancer Cells
Source: Int J Mol Sci. 2019 Jul 8;20(13):3344. doi: 10.3390/ijms20133344 (PMC6651286; doi:10.3390/ijms20133344)
Supplement: Supplementary file 1 [file ijms-20-03344-s001.pdf]

Supplementary materials

Table S1. siRNA sequences

Target gene	Sense	Antisense
Control	UUCUCCGAACGUGUCACGUTT	ACGUGACACGUUCGGAAATT
NRF3#1	GGAUCAAAGUGAUUCUGAUTT	AUCAGAAUCACUUUGAUCCAA
NRF3#2	GCAAAGAAGGAAACUCUUATT	UAAGAGUUUCCUUCUUUGCUU
β-catenin#1	UGGUUGCCUUGCUCAACAATT	UUGUUGAGCAAGGCAACCATT
β-catenin#2	AGCUGAUAUUGAUGGACAGTT	CUGUCCAUCAAUAUCAGCUTT
TCF4#1	GAUGGAAGCUUACUAGAUUTT	AAUCUAGUAAGCUUCCAUCTT
TCF4#2	CAAUGAAUCAGAAACGACGAAUTT	AUUCGUUUCUGAUUCAUUGTT

Table S2. Primer sequences

qRT-PCR

Gene	Forward primer	Reverse primer
β-Actin	CCAACCGCGAGAACA	CCAGAGGCGTACAGGG
NRF1	TGGAACAGCAGTGGCAAGATCTCA	GGCACTGTACAGGATTTCACTTGC
NRF2	TACTCCCAGGTTGCCCACA	CATCTACAAACGGGAATGTCTGC
NRF3	CTGACTGGGAGGCAGAAAAG	TCAGGCTGTGATGAAAGCAA
β-catenin	TGAGGACAAGCCACAAGATTAC	TCCACCAGAGTGAAAAGAACG
TCF4	CCGACGTAGACCCCAAAACA	ATCCTAGCGGATGGGGGATT
LEF1	ACAGATCACCCCACCTCTTG	TGATGGGAAAACCTGGACAT
GLUT1	CTCATCGCTGTGGTGCTG	GGCCGTGTTGACGATACC
Gapdh	CAGAACTACATCCCTGCATCC	CCACCTTCCTGATGTCATCA
Nrf1	AGCCTGCTTTTCCTTGCTAC	CATACAATATGGCACCCAGT
Nrf2	CAAGACTTGGGCCACTTAAAAG	GTAAGGCTTTCCATCCTCATCAC
Nrf3	GCTAAGAGAAAAGACCGAAGCTG	CTGCTCAGAAAAGGAATGTC
Nqo1	AGCTGGAAGCTGCAGACCTG	CCTTTCAHAATGGCTGGCA
Axin2	AAGAGAAGCGACCCAGTCAA	CTGCGATGCATCTCTCTCTG
c-Myc	GCAGGGTCCTGAAGCAGA	GTGTCCGCCTCTTGTCGT
Lgr5	GGCACGTAGCTGATGTGGT	TGCAGAAACAACCAGCTGAGA
Cd44	GGACTTTGCCTCTTGCAGTT	CTGTAGCGGCCATTTTTCTC
Glut1	CTCATCGCTGTGGTGCTG	GGCCGTGTTGACGATACC
Gclm	TGGAGCAGCTGTATCAGTGG	AAATCTGGTGGCATCACACA
xCt	GGTTGCCCTTGGCTTTTT	CAGATTGCAAGGGGGATG

ChIP primer

Target region	Forward primer	Reverse primer
NRF3_WRE	GGATAAGTGTGACCTTAG	GGCTAGTGGGATGCATGG
GLUT1_ARE1	TTCATCACGGAGGAGCTG	GCTCCTGTGGCTCTCAAC
GLUT1_ARE2	CGGGCTGGGAAAAGACTC	CCCTGCTATCCCCTCTTG
GLUT1_ARE3	CATGCCCAGCAAACTTGG	CCGATGCTGAGGAGCAAG

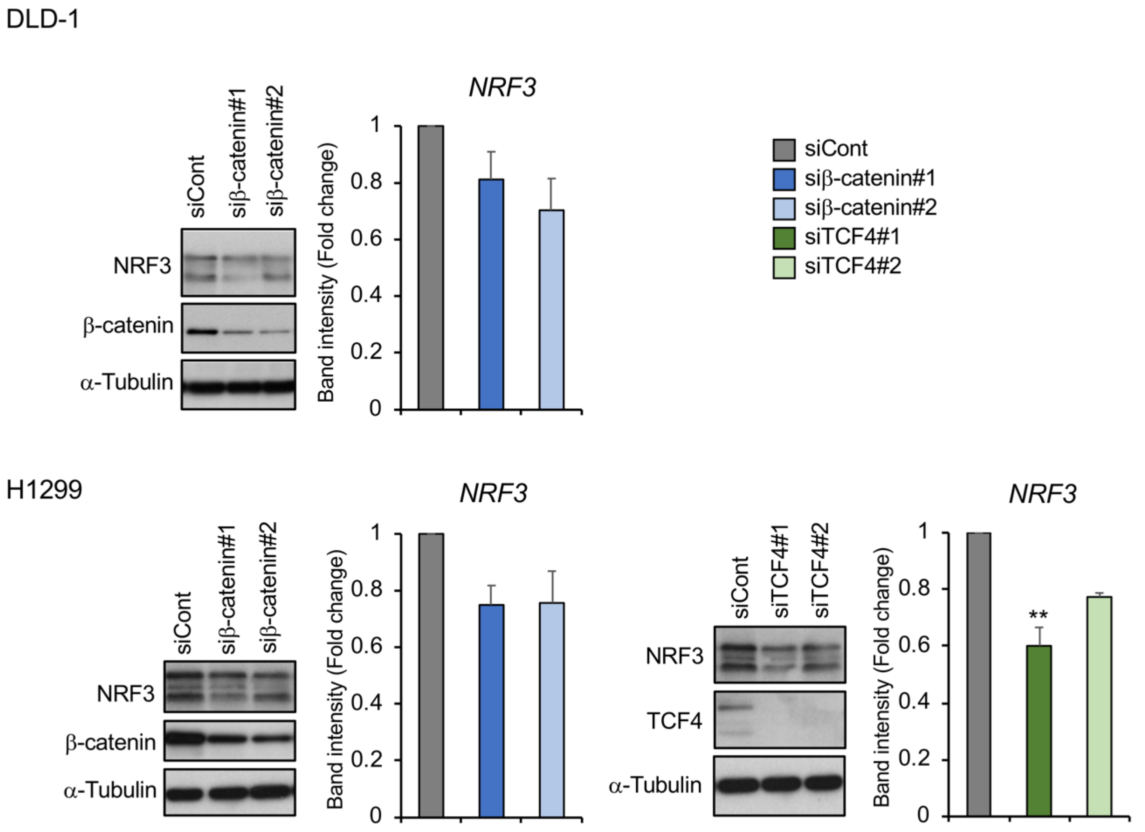

Supplementary Figure S1. Knockdown of either β-catenin or TCF4 exhibits moderately reduction of NRF3 protein levels in DLD-1 and H1299 cells. Western blot analyses using the indicated antibodies were performed. Protein expression were normalized to α-Tubulin protein expression. The data are presented as the mean ± SD (n=3). ** p<0.01 (ANOVA-Tukey).

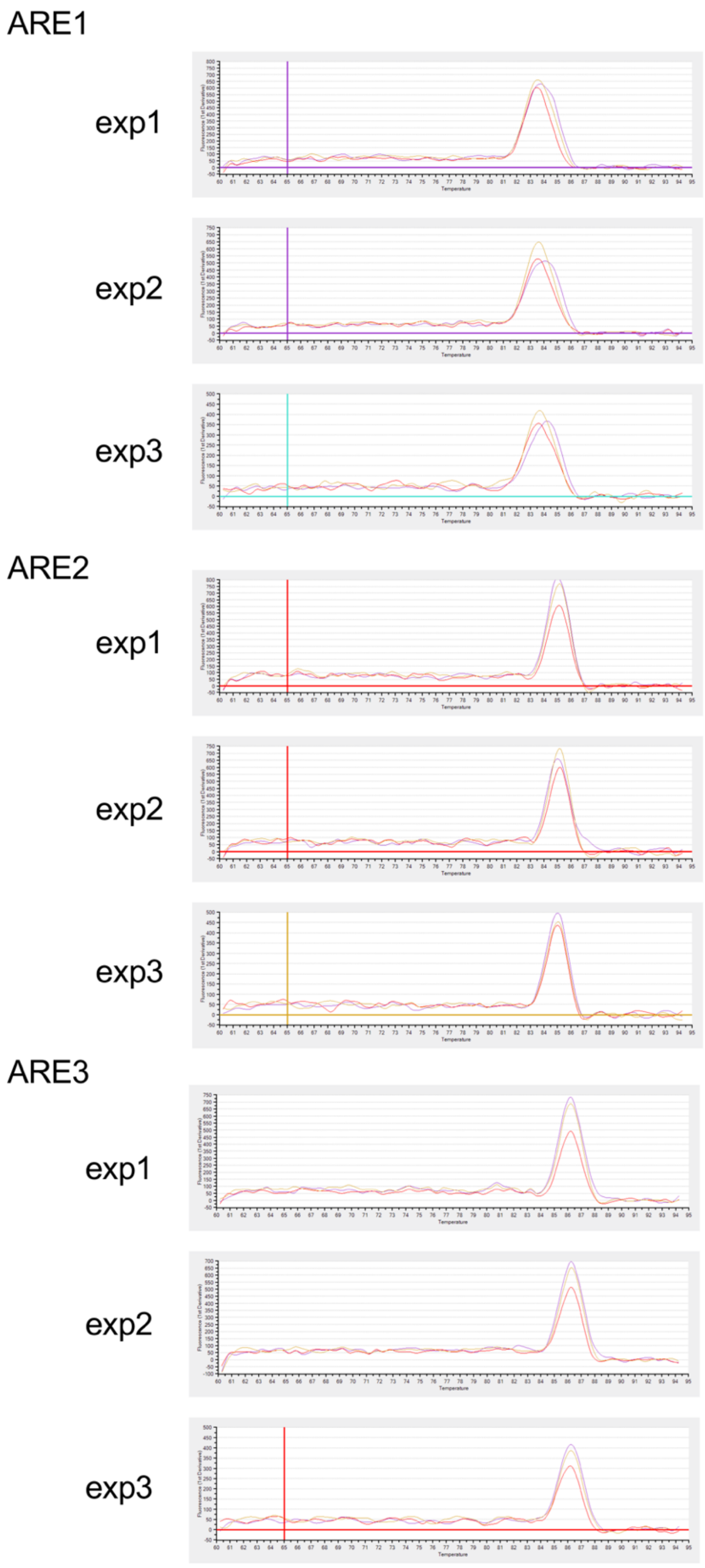

Supplementary Figure S2. Melting curves of qRT-PCR in the ChIP analysis of Figure 4C, indicating the specificity of the reaction.

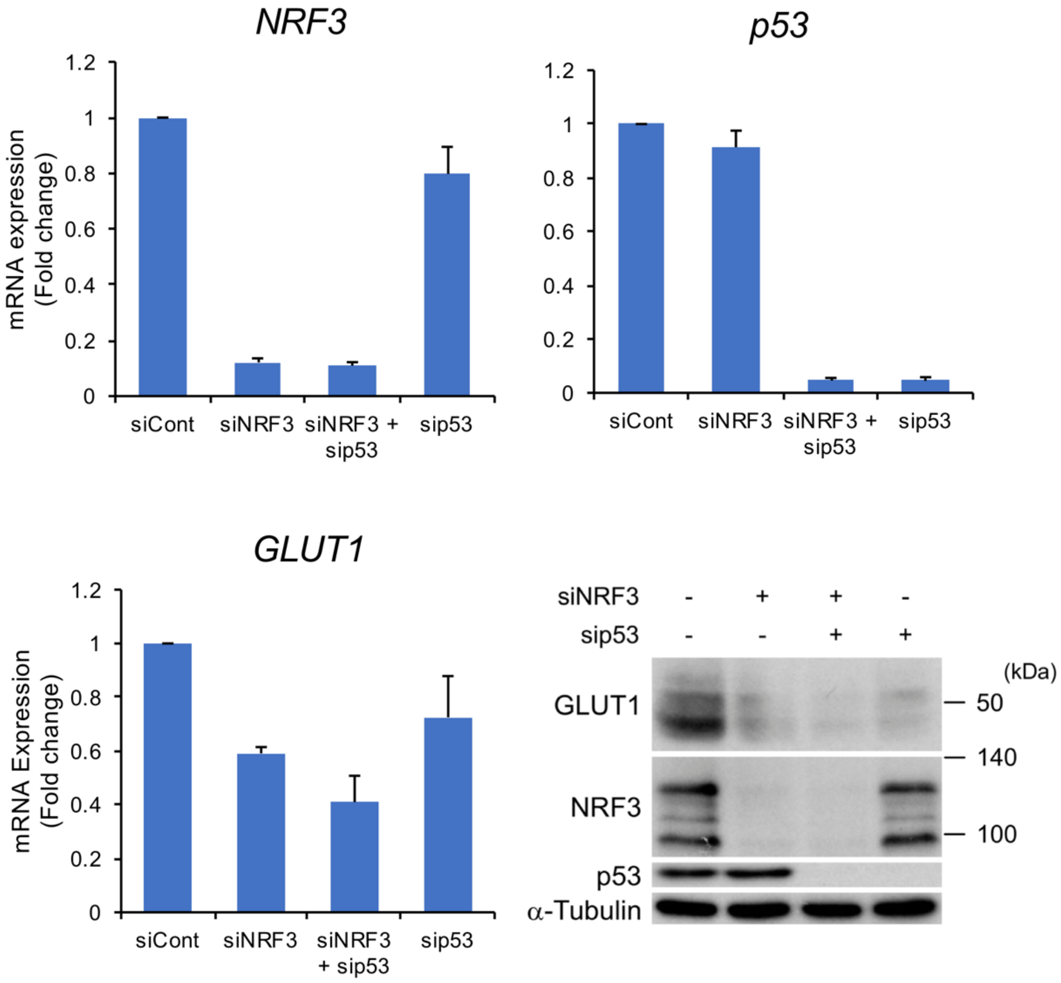

Supplementary Figure S3. The NRF3-mediated *GLUT1* gene expression does not require the p53 reduction. Knockdown of *p53* and/or *NRF3* was performed in HCT116 cells. mRNA expression was normalized to *β-actin* mRNA. The data are presented as the mean ± SD (n=3).
